# Extracellular biosynthesis of gadolinium oxide (Gd_2_O_3_) nanoparticles, their biodistribution and bioconjugation with the chemically modified anticancer drug taxol

**DOI:** 10.3762/bjnano.5.27

**Published:** 2014-03-07

**Authors:** Shadab Ali Khan, Sanjay Gambhir, Absar Ahmad

**Affiliations:** 1 Biochemical Sciences Division, CSIR-National Chemical Laboratory, Pune 411008, India; 2 Department of Nuclear Medicine, SGPGIMS, Lucknow-226014(U.P), India

**Keywords:** bioconjugation, biodistribution, gadolinium oxide, *humicola sp*, transmission electron microscopy

## Abstract

As a part of our programme to develop nanobioconjugates for the treatment of cancer, we first synthesized extracellular, protein-capped, highly stable and well-dispersed gadolinium oxide (Gd_2_O_3_) nanoparticles by using thermophilic fungus *Humicola sp.* The biodistribution of the nanoparticles in rats was checked by radiolabelling with Tc-99m. Finally, these nanoparticles were bioconjugated with the chemically modified anticancer drug taxol with the aim of characterizing the role of this bioconjugate in the treatment of cancer. The biosynthesized Gd_2_O_3_ nanoparticles were characterized by UV–vis spectroscopy, transmission electron microscopy (TEM), X-ray diffraction (XRD) and X-ray photoemission spectroscopy (XPS). The Gd_2_O_3_–taxol bioconjugate was confirmed by UV–vis spectroscopy and fluorescence microscopy and was purified by using high performance liquid chromatography (HPLC).

## Introduction

Gadolinium oxide nanoparticles are very important as nuclear, electronic, laser, optical, catalyst and phosphor materials [1–4]. Many organic compounds use Gd_2_O_3_ for their dimerization [2]. Moreover, it is used in imaging plate neutron detectors, as neutron convertor [2–3], as additives in UO_2_ fuel rods for nuclear reactors [2], and as an additive in ZrO_2_ to enhance its toughness [3–4]. Gd_2_O_3_ has several potential applications in biomedicine, too. For example, it is used in magnetic resonance imaging, since it exhibits superparamagnetism and involves T1 relaxation, and can be useful as a multimodal contrast agent for in vivo imaging [5]. It can also be easily doped with other lanthanides and exploited as a fluorescent tag, thus replacing other fluorescent organic molecules.

Gadolinium oxide nanoparticles are also employed in site-specific drug delivery systems for cancer therapy. Gadolinium compounds are used in neutron capture therapy (NCT) as an alternative for boron-10 [6–7]. NCT is mainly associated with tumor-specific delivery systems and involves the production of localized cytotoxic radiations by a non-radioactive nuclide delivered to tumor cells. These cytotoxic radiations, which are produced by the irradiation of a radioactive nuclide by thermal or epithermal neutrons, will eventually destroy the tumorous/cancerous cells. High energy gamma rays and low-energy Auger and internal conversion electrons emitted during the therapy are mainly responsible for the tumor killing efficiency of Gd-NCT [8]. Gd-157 not only requires shorter neutron irradiation time but also has a large neutron capture cross section area than boron-10, so that it is an ideal substitute for boron-10.

As far as synthesis methods for Gd_2_O_3_ nanoparticles are concerned, the chemical and physical protocols are limited, and its synthesis is seldom encountered in literature. The most common methods are the thermal decomposition of precursor salts, mechanochemical processing, milling and calcinations [9–11]. Unfortunately, these methods give agglomerated particles, occur at high temperatures, and employ harsh environments, thus rendering it difficult to find any usage of Gd_2_O_3_ nanoparticles in biomedical applications. Our group has already reported the biological synthesis of zirconia, titania, silica and CuAlO_2_ nanoparticles [12–14]. In this work, we employed a fungus based approach for the synthesis of this material for the first time. We show that the thermophilic fungus *Humicola sp.* can be used for the synthesis of Gd_2_O_3_ nanoparticles at 50 °C. Since Gd_2_O_3_ nanoparticles have proved their value in site-specific drug delivery systems for cancer therapy, we extended the work of biosynthesis of Gd_2_O_3_ nanoparticles to bioconjugation with taxol. Bioconjugation of taxol with gold and iron oxide nanoparticles has also been reported [15–16]. Taxol is one of the most important anticancer drugs used for breast, ovarian and lung cancers [17–18]. The potent anticancer effect of taxol is mainly attributed to its mechanism of action. It stabilizes microtubules by preventing their depolymerization [19–20]. However, taxol is a hydrophobic drug and less specific to certain tumors due to its low solubility in water. To counter these problems, we carried out the bioconjugation of chemically modified taxol with biocompatible Gd_2_O_3_ nanoparticles.

## Experimental

### Materials

Gadolinium chloride (GdCl_3_) and sodium carbonate were obtained from Sigma Aldrich. Malt extract, yeast extract, glucose and peptone were obtained from HiMedia and used as received.

### Methods

The thermophilic fungus *Humicola sp.* was cultured and maintained by us as described previously [21].

### Biosynthesis of gadolinium oxide nanoparticles

The harvested mycelial mass weighing 20 g [21] was suspended in 100 mL of 10^−3^ M aqueous gadolinium chloride solution in a 250 mL Erlenmeyer flask at pH 9. The whole mixture was put onto a shaker at 50 °C (200 rpm) and maintained in the dark.

### Characterization of gadolinium oxide nanoparticles

#### UV–vis spectroscopy

To check the synthesis of gadolinium oxide nanoparticles, the mixture was monitored by periodic sampling of aliquots (2 mL) of the aqueous component. The measurement was carried out on a Shimadzu dual-beam spectrophotometer (model UV-1601 PC) operated at a resolution of 1 nm.

#### Transmission electron microscopy (TEM)

TEM analyses of gadolinium oxide nanoparticles were carried out on a JEOL model 1200 EX operated at 80 kV. Samples were prepared by drop-casting the particles (suspended in water) on carbon coated copper grids.

#### High resolution (HR)-TEM

HR-TEM analysis was carried out on a TECHNAI G2 F30 S-TWIN instrument operated at an acceleration voltage of 300 kV with a lattice resolution of 0.14 nm and a point image resolution of 0.20 nm. A sample was prepared by drop-casting the particles (suspended in water) on carbon coated copper grid. The selected area electron diffraction (SAED) pattern analysis was carried out on the same grid.

#### X-ray diffraction (XRD)

X-ray diffraction (XRD) measurements of biosynthesized Gd_2_O_3_ nanoparticles were carried out by coating the Gd_2_O_3_ powder on a glass substrate on a Philips X’PERT PRO instrument equipped X’celerator. Iron-filtered Cu Kα radiation (λ = 1.5406 Å) was used and the sample was scanned by using X’celerator with 121 active channels. XRD patterns were recorded in the 2θ range of 10–80° with a step size of 0.02° and a time of 5 seconds per step at 40 kV voltage and a current of 30 mA.

#### X-ray photoemission spectroscopy (XPS)

XPS of Gd_2_O_3_ nanoparticles powder was carried out on a VG microtech ESCA (XPS) 3000 spectrometer. The base pressure during XPS analysis was 1 × 10^−9^ Torr and Mg Kα X-ray radiation (1253.6 eV) at a power of 200 watts was used. The binding energy of Au (4f7/2) at 84.0 ± 0.1 eV was used to calibrate the binding energy scale of the spectrometer. Any charging shift produced in the spectrum was corrected by referencing to the C (1s) position (284.6 eV) Background correction of core level spectra was performed by using the Shirley algorithm. The chemically distinct species were resolved by a nonlinear least square fitting procedure.

### Radiolabelling and biodistribution studies

#### Radiolabelling of gadolinium oxide (Gd_2_O_3_) nanoparticles with Tc-99m

To fabricate Tc-99m–Gd_2_O_3_ nanoparticles, 10 mg of Gd_2_O_3_ nanoparticles were dissolved in 1 mL of distilled water, to which 100 μg of SnCl_2_·2H_2_O was added, and the pH was brought to 6.5. A 0.22 μm membrane filter was employed to filter the contents into a sterile vial to which approximately 2 mCi of Tc-99m was added and the mixture was incubated for 10 min. The instant thin layer chromatography (ITLC) method was used to determine the percentage of radiolabeling [22].

#### Radiochemical purity (RCP)

ITLC with silica gel coated fiber sheets was used to estimate the radiochemical purity of Tc-99m with Gd_2_O_3_ nanoparticles employing 100% acetone and 0.9% saline as the mobile phase. To the ITLC-SG strip, 2–3 μL of the radiolabeled complex was applied at a point 1 cm from the end and allowed to run for approximately 10 cm. ITLC as the stationary phase and pyridine/acetic acid/water (3:5:1.5 v/v) as the mobile phase were used in determining the amount of reduced/hydrolyzed Tc-99m. A radioactivity well counter (ECIL) was employed in determining the radioactivity distribution over the strip. The fraction of radioactivity remaining at the origin determined the radiochemical purity (RCP), which was designated as % RCP.

#### Biodistribution of radiolabelled nanoparticles

A male Sprauge Dawley rat weighing 180–220 g was chosen to evaluate the localization of the labeled complex. The Tc-99m–Gd_2_O_3_ nanoparticles of 14.8 MBq were administered into the rat through its penile vein. The biodistribution studies of these nanoparticles were conducted 45 min post-injection.

### Bioconjugation of taxol with Gd_2_O_3_ nanoparticles

#### Materials

Glutaric anhydride, pyridine, 1,1’-carbonyldiimidazole (CDI), *tert*-butyldimethylsilyl chloride, imidazole, dimethylformamide (DMF), succinic anhydride, 4-dimethylaminopyridine, 1-ethyl-3-(3-dimethylaminopropyl)carbodiimide (EDC), 3-nitro-L-tyrosine ethyl ester hydrochloride (NTEE), 1-hydroxybenzotriazol (HBT), 2-(*N*-morpholino)ethanesulfonic acid (MES) and 2-[4-(2-hydroxyethyl)piperazin-1-yl]ethanesulfonic acid (HEPES) were purchased from Sigma, and HPLC grade solvents (acetonitrile, chloroform, etc) were purchased from Merck.

#### Methods/modification of taxol

**Synthesis of 2’-glutaryltaxol:** 2’-Glutaryltaxol was prepared by reacting 10 mg of taxol, dissolved in 1.2 mL of pyridine, with 140 mg of glutaric anhydride [23]. The reaction was carried out at room temperature for about 2 h and was monitored on TLC by using a mobile phase of chloroform/acetonitrile (7:3). After the incubation period, the solvent was evaporated under high vacuum, and the residue was washed twice with water. The obtained product was precipitated by using acetone and further purified by preparative TLC by using the mobile phase chloroform/acetonitrile (7:3).

**Synthesis of 2’-glutarylhexanediamine taxol:** The recovered 2’-glutaryltaxol from the preparative TLC was solvent dried and dissolved in 100 µL of dry acetonitrile, 5 µmol of 1,1’-carbonyldiimidazole (CDI) was added, and it was heated at 45 °C for about 15 min. After the reaction mixture was at room temperature, 5 µmol of 1,6-hexanediamine·2HCl was added, and it was incubated at room temperature for 1 h. The reaction was monitored on TLC and purified as described above.

#### Estimation of free carboxyl groups on Gd_2_O_3_ nanoparticles and bioconjugation with taxol

Biologically synthesized Gd_2_O_3_ nanoparticles have a natural protein coat. The carboxyl groups present on this protein capped nanoparticles were targeted to couple with the free amino group present in 2’-glutarylhexanediamine taxol and estimated by the following procedure:

The total reaction mixture of 3 mL containing 100 μg of Gd_2_O_3_ nanoparticles in 50 mM MES/HEPES buffer (75:25 v/v) pH 6.0, 50 mM EDC and 30 mM NTEE was incubated at 30 °C for 45 min. Subsequently, the reaction was terminated with the addition of 1 mL of 10% TCA, and the precipitated Gd-peptide complex was collected by centrifugation, washed extensively with chilled acetone, air-dried and dissolved in 1 mL of 100 mM NaOH. The number of nitrotyrosyl groups was determined spectrophotometrically at 430 nm by using a molar absorption coefficient of 4600 M^−1^ cm^−1^. 2’-Glutarylhexanediamine taxol (400 µg) was dissolved in anhydrous DMF (300 µL), and EDC (1.2 µmol, 1.1 equiv) along with 1-hydroxybenzotriazol (HBT) (4 µmol, 2.2 equiv). The reaction mixture was stirred at room temperature for about 1 h, and a solution of Gd_2_O_3_ nanoparticles in phosphate buffer of pH 7.2 was added. After stirring for 12 h at room temperature, the reaction mixture was concentrated under a high vacuum. Further purification of the 2’-glutarylhexanediamine-taxol–Gd_2_O_3_ bioconjugate was carried out by HPLC.

### Characterization of Gd_2_O_3_–taxol bioconjugate

#### UV–vis spectroscopy

The UV–vis spectroscopic analysis of Gd_2_O_3_–taxol bioconjugate was carried out on a Shimadzu dual-beam spectrophotometer (model UV-1601 PC) operated at a resolution of 1 nm.

#### Fluorescence microscopy

Fluorescence measurements of Gd_2_O_3_–taxol bioconjugate were carried out by using a Perkin Elmer LS-50B spectrofluorimeter with a slit width of 7 nm for both monochromators and a scan speed of 100 nm/min.

#### Purification of Gd_2_O_3_–taxol bioconjugate by HPLC

The bioconjugate from other chemical contaminants was purified by HPLC (Waters model 2489 with UV–vis detector) by using Acetonitrile 5–95% on a C_18_ symmetry column. The compounds eluted from the columns were detected at 227 nm and 325 nm by using a dual wavelength detector.

## Results and Discussion

### UV–vis spectroscopy

**Figure 1:**
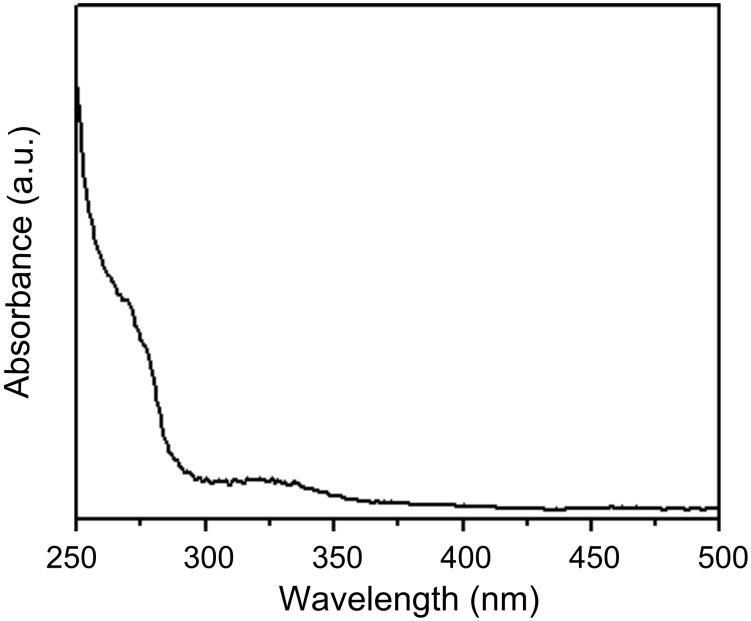
UV–vis spectrum of biosynthesized gadolinium oxide nanoparticles solution after 96 h of reaction with the fungal biomass.

Figure 1 shows the UV–vis spectrum of biosynthesized Gd_2_O_3_ nanoparticles after 96 h of reaction with the fungus *Humicola sp.* The UV–vis spectrum of biosynthesized Gd_2_O_3_ nanoparticles indicates two regions of absorption at 270 nm and 325 nm. It is well established that the absorption edge at ca. 270 nm arises due to electronic transitions in the delocalized π-electrons present in the indole ring of aromatic amino acids such as tryptophan, tyrosine and to some extent phenylalanine residues, which are present in the proteins moiety [24]. These residues of proteins may be secreted in the solution by the fungus *Humicola sp.* in response to the stress conditions encountered by the fungus in the presence of GdCl_3_. Some of these amino acid residues constitute the protein layer, which can cap the nanoparticles. As soon as GdCl_3_ gets dissolved in water along with fungal biomass, it ionizes to Gd^3+^ and 3Cl^−^. The Gd^3+^ ions are then attracted toward anionic proteins, which are secreted by the fungus in solution. Certain reductase enzymes present in the anionic protein fraction act on Gd^3+^ and convert it to Gd^2+^. Oxidase enzymes, which are also secreted by the fungus in the solution mixture, act on these Gd^2+^ ions resulting in the formation of Gd_2_O_3_ nanoparticles. Hence, complementary actions of oxidases and reductases, which are secreted by the fungus *Humicola sp.*, play a very vital role in the formation of Gd_2_O_3_ nanoparticles. Biosynthesized Gd_2_O_3_ nanoparticles show an absorption peak at ca. 325 nm. This edge may be attributed to d–d and f–f transitions occurring in mixed valence transition metal compounds [24].

**Figure 2:**
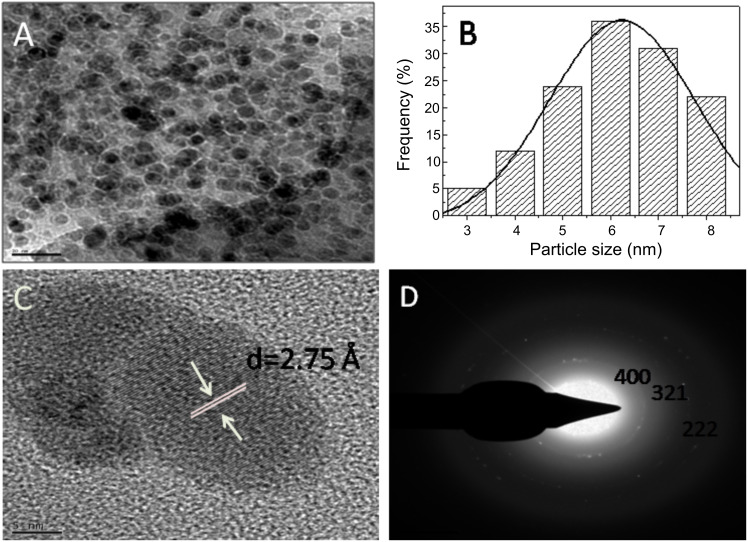
(A) TEM micrograph recorded from drop-cast films of Gd_2_O_3_ nanoparticle solution formed by the reaction of GdCl_3_ with the fungal biomass of *Humicola sp.* for 96 h. (B) Particle size distribution determined from TEM microgaph. (C) HR-TEM image of Gd_2_O_3_ nanoparticles showing inter planar distance. (D) Selected area electron diffraction (SAED) pattern recorded from the Gd_2_O_3_ nanoparticles.

Figure 2A represents the transmission electron microscopic (TEM) image of the fungus–GdCl_3_ reaction mixture after 96 h of reaction. The particles are irregular in shape, presenting an overall quasi-spherical morphology. Particle size distribution analysis of Gd_2_O_3_ nanoparticles confirmed that the nanoparticles are in the range of 3–8 nm with an average size of 6 nm (Figure 2B). The interplanar distance of Gd_2_O_3_ nanoparticles was estimated to be 2.75 Å and corresponds to plane {400} of Gd_2_O_3_ nanoparticles (Figure 2C). Selected area electron diffraction (SAED) analysis (Figure 2D) of the biosynthesized Gd_2_O_3_ nanoparticles shows that the nanoparticles are crystalline in nature. Diffraction spots could be well indexed with the cubic structure of Gd_2_O_3_ nanoparticles and the obtained three rings corresponding to the {400}, {321} and {222} planes of Gd_2_O_3_ and are in good agreement with the reported values [25].

**Figure 3:**
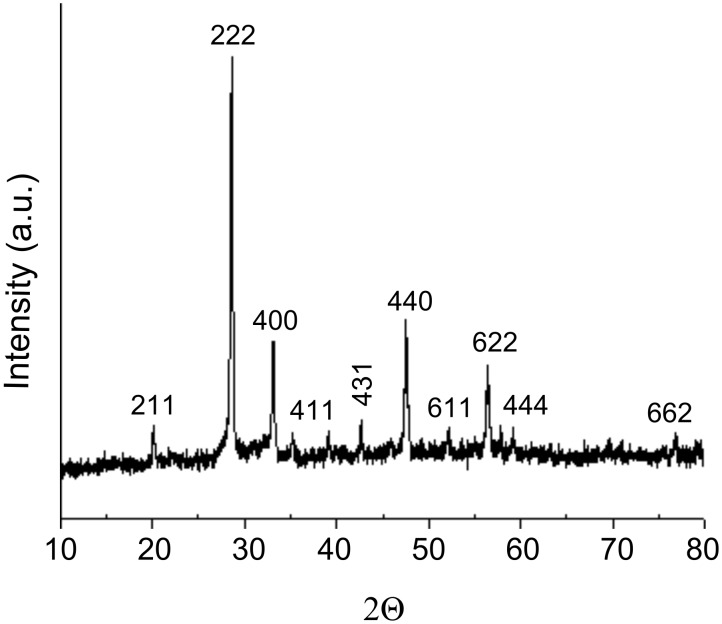
XRD measurements of biosynthesized Gd_2_O_3_ nanoparticles.

Figure 3 displays the X-ray diffraction (XRD) analysis of the biosynthesized gadolinium oxide nanoparticles carried out by depositing Gd_2_O_3_ powder on Si substrate. The XRD measurements show intense peaks corresponding to the planes {211}, {222}, {400}, {411}, {431}, {440}, {611}, {622}, {444} and {662}. The peak position and 2θ values agree with those reported for gadolinium oxide nanoparticles [25].

**Figure 4:**
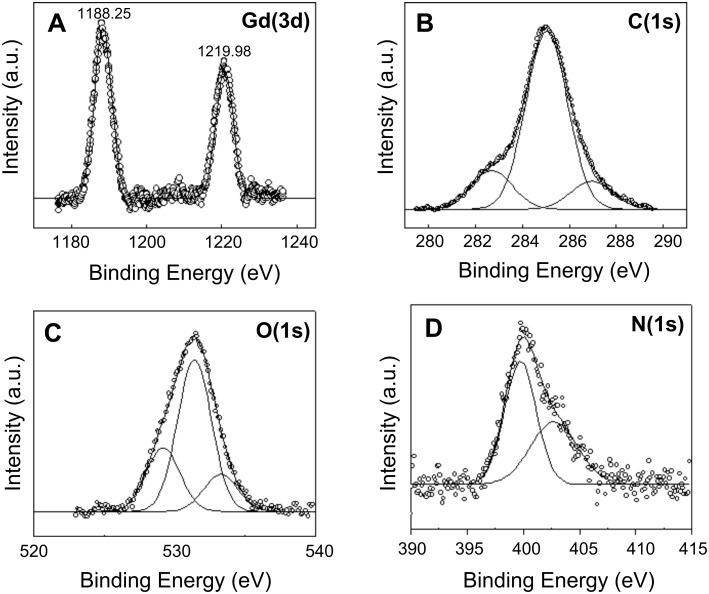
XPS data showing the (A) Gd(3d), (B) C(1s), (C) O(1s) and (D) N(1s) core level spectra recorded from biosynthesized Gd_2_O_3_ nanoparticles film cast onto a Si substrate. The raw data are shown in the form of symbols, while the chemically resolved components are shown as solid lines and are discussed in the text.

Figure 4 represents the XPS analysis of biosynthesized Gd_2_O_3_ nanoparticles. The Gd(3d) spectrum of Gd_2_O_3_ nanoparticles coated onto a Si substrate is shown in Figure 4A. The Gd(3d) level consists of a spin orbit split doublet, with the Gd(3d5*/*2) and Gd(3d3*/*2) peaks at 1188.25 and 1219.98 eV, respectively. The line shape and peak positions are in good agreement with earlier published data on Gd_2_O_3_ nanoparticles, confirming that the sample consists of Gd_2_O_3_ [26]. The C(1s) spectrum in Figure 4B shows three different peaks at 282.67, 285.03 and 287.01 eV and can be attributed to α-carbon, hydrocarbon chains and –COOH of the proteins associated with Gd_2_O_3_ nanoparticles. Figure 4C represents the O(1s) spectrum which shows three distinct peaks. The peak at 531.30 eV corresponds to the oxygen in the Gd_2_O_3_ nanoparticles [26], whereas peaks at 529.18 and 533.26 eV originate from the oxygen in the carboxyl groups of proteins associated with Gd_2_O_3_ nanoparticles. Figure 4D shows the N(1s) core level spectra that could be decomposed into two chemically distinct components centered at 399.6 and 402.5 eV and can be attributed to the neutral amino group NH_2_ and N atoms present in amide bonds of protein capping Gd_2_O_3_ nanoparticles [26]. These signatures of carbon and oxygen arising from proteins exposed a prominent role of proteins and enzymes in the reduction and capping of Gd_2_O_3_ nanoparticles.

**Figure 5:**
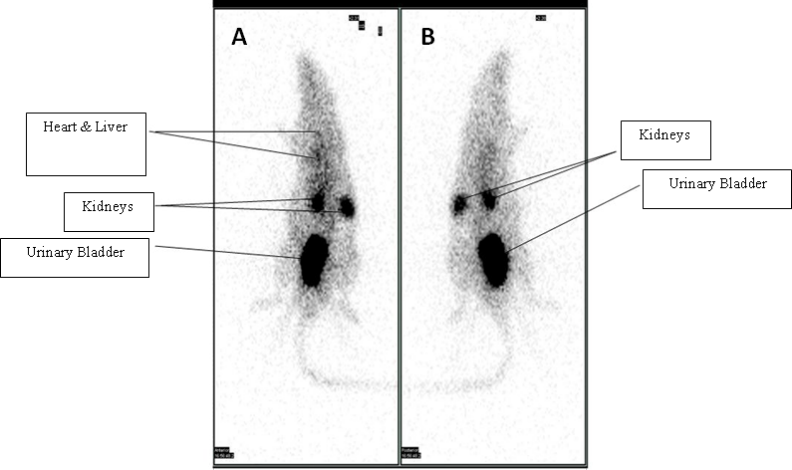
Gamma scintigraphic image of the biodistribution of Tc-99m–Gd_2_O_3_ nanoparticles in a rat showing a dorsal (A) and a ventral (B) view.

Figure 5 represents a dorsal (A) and ventral (B) view of the biodistribution and gamma scintigraphic image of Tc-99m–Gd_2_O_3_ nanoparticles in a normal rat . We also studied the complex formation on the basis of a chromatographic analysis, and the radiolabelling efficiency was found to be more than 90%. The localization and biodistribution of Tc-99m–Gd_2_O_3_ nanoparticles in a healthy rat over time was determined by gamma camera imaging. The study clearly indicates the biodistribution of the complex (Tc-99m–Gd nanoparticle), these Gd_2_O_3_ nanoparticles were taken up in the liver, heart, kidneys and cleared through urine within 45 min.

**Figure 6:**
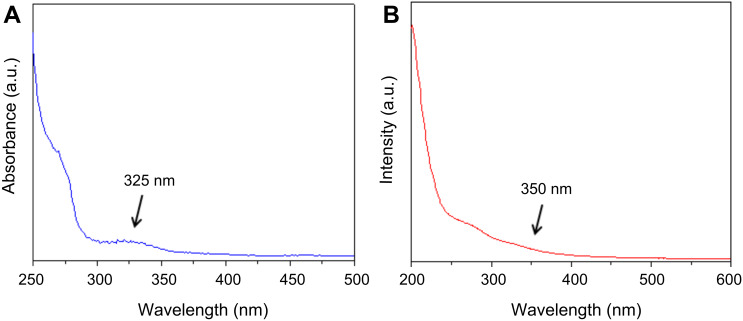
UV–vis spectroscopy of (A) Gd_2_O_3_ nanoparticles showing a peak at 325 nm and (B) Gd_2_O_3_–taxol bioconjugate showing a shoulder at 350 nm.

Figure 6A and 6B show the UV–vis analyses of gadolinium oxide nanoparticles and Gd_2_O_3_–taxol bioconjugate, respectively. Gadolinium oxide (Gd_2_O_3_) nanoparticles showed a peak at ca. 325 nm (Figure 6A), which after conjugation with taxol red shifted to 350 nm (Figure 6B). This type of red-shifting after conjugation has been explained by several reports [27–28]. Since the conjugation of a drug with nanoparticles causes the drug to be slightly heavier, conjugates tend to absorb at higher wavelengths.

**Figure 7:**
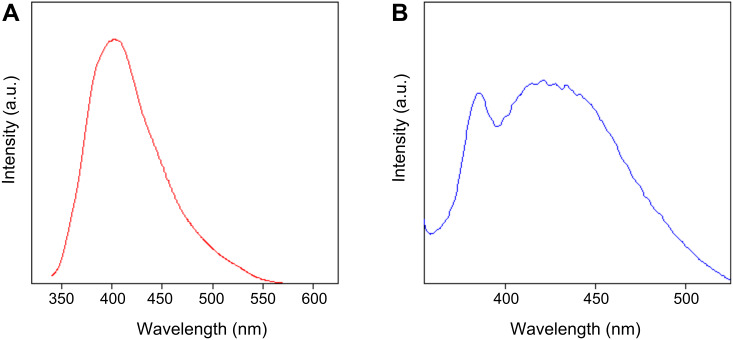
(A) Fluorescence spectra of Gd_2_O_3_ nanoparticles excited at 320 nm giving emission at 400 nm and (B) Gd_2_O_3_–taxol bioconjugate excited at 320 nm giving emission at 440 nm.

Figure 7A represents fluorescence spectra of Gadolinium oxide (Gd_2_O_3_) nanoparticles and Figure 7B Gd_2_O_3_–taxol bioconjugate. Both samples were excited at 320 nm. Gadolinium oxide (Gd_2_O_3_) nanoparticles gave a sharp emission at 400 nm, whereas Gd_2_O_3_–taxol bioconjugate gave an emission spectrum with λ_max_ at 440 nm. This red-shifting of λ_max_ occurs due to the coupling of gadolinium oxide (Gd_2_O_3_) nanoparticles with taxol.

**Figure 8:**
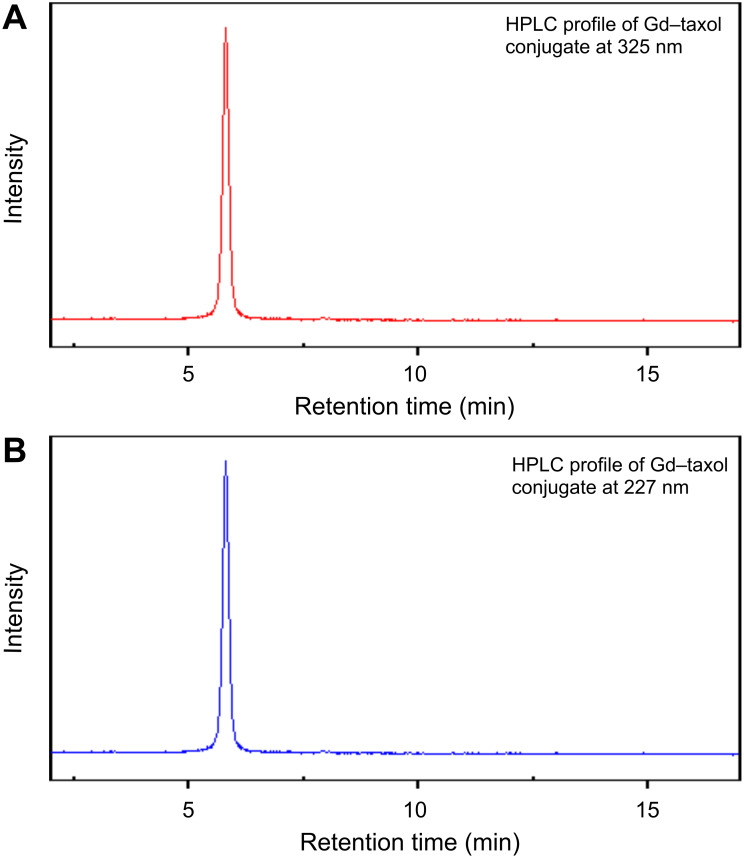
HPLC profile of Gd_2_O_3_–taxol bioconjugate showing absorbance at (A) 325 nm and (B) 227 nm.

Figure 8 shows the HPLC profile of Gd_2_O_3_–taxol bioconjugate detected at 325 nm (Figure 8A) and 227 nm (Figure 8B), which are attributed to the absorption maxima of Gd_2_O_3_ nanoparticles and taxol, respectively. From the figure, it is very clear that Gd_2_O_3_–taxol bioconjugate emerged as a single peak at both wavelengths and with the same retention time, thus confirming the conjugation of taxol with Gd_2_O_3_ nanoparticles.

## Conclusion

We demonstrated a simple biological protocol for the synthesis of gadolinium oxide nanoparticles, studied their biodistribution, and bioconjugated these nanoparticles with the chemically modified anticancer drug taxol. This particular bioconjugation may result in an enhancement of the hydrophilicity of taxol and may render it more potent in killing tumor/cancer cells. We believe that this work could pave the way for nanosized drug delivery applications for the treatment of cancer.
